# Electronic Prediction of Chemical Contaminants in Aroma of Brewed Roasted Coffee and Quantification of Acrylamide Levels

**DOI:** 10.3390/foods13050768

**Published:** 2024-03-01

**Authors:** Gema Cascos, Ismael Montero-Fernández, Jhunior Abrahan Marcía-Fuentes, Ricardo S. Aleman, Antonio Ruiz-Canales, Daniel Martín-Vertedor

**Affiliations:** 1 Technological Institute of Food and Agriculture (CICYTEX-INTAEX), Junta of Extremadura, Avda. Adolfo Suárez, s/n, 06007 Badajoz, Spain; gcascosc01@educarex.es; 2 Department of Chemical Engineering and Physical Chemistry, Area of Chemical Engineering, Faculty of Sciences, University of Extremadura, Avda. de Elvas, s/n, 06006 Badajoz, Spain; ismonterof@unex.es; 3 Faculty of Technological Sciences, Universidad Nacional de Agricultura, Catacamas 16201, Olancho, Honduras; jmarcia@unag.edu.hn; 4 School of Nutrition and Food Sciences, Louisiana State University Agricultural Center, Baton Rouge, LA 70803, USA; rsantosaleman@lsu.edu; 5 Engineering Department, Polytechnic High School of Orihuela, Miguel Hernández University of Elche, 03312 Orihuela, Spain; acanales@umh.es

**Keywords:** roasted coffee, sensory analysis, electronic nose, acrylamide, 5-hydroxymethylfurfural, volatile compounds

## Abstract

The aim of this research was to apply an electronic device as indirect predictive technology to evaluate toxic chemical compounds in roasted espresso coffee. Fresh coffee beans were subjected to different thermal treatments and analyzed to determine volatile organic compounds, content of acrylamide and 5-hydroxymethylfurfural, sensory characteristics and electronic nose data. In total, 70 different volatile compounds were detected and grouped into 15 chemical families. The greatest percentage of these compounds were furans, pyrazines, pyridines and aldehydes. The positive aroma detected had the intensity of coffee odor and a roasted aroma, whereas the negative aroma was related to a burnt smell. A linear relationship between the toxic substances and the sensory defect was established. A high sensory defect implied a lower content of acrylamide and a higher content of 5-hydroxymethylfurfural. Finally, electronic signals were also correlated with the sensory defect. This relationship allowed us to predict the presence of these contaminants in the roasted coffee beverage with an indirect method by using this electronic device. Thus, this device may be useful to indirectly evaluate the chemical contaminants in coffee beverages according to their sensory characteristics.

## 1. Introduction

Coffee is one of the most commercialized beverages worldwide. Its fruit belongs to the Coffea genus made up of some 100 species of which the most commercially used are the Arabica (*Coffea arabica* L.) and Robusta (*Coffea canephora* L.) varieties [1]. Coffee production in 2022 saw an increase of 4.2%, totaling 175.6 million bags. Considering this demand, producers were expected to increase coffee production in the 2023 campaign by 1.7% [2]. The coffee beans of the Arabica variety surpass those of the Robusta variety in terms of quality, with Arabica presenting greater aromatic complexity [3]. The quality of coffee depends largely on the aroma obtained in the roasting process, whose composition of volatile compounds is complex, with more than 800 compounds identified [4]. Thus, roasting times must be precisely controlled as they generate different degrees of roasting that vary from a very light to a very dark color and influence the aroma generated by the different types of volatile compounds [5].

A wide and complicated pathway of reactions is formed in the coffee roasting process. In some cases, this process can produce positive reactions and in others, negative reactions can arise from the transformation of precursors. The principal precursors are sugars, amino acids, unsaturated fatty acids, fats and carotenoids [6]. The Maillard and Strecker reactions represent the main reactions of this type in which proteins, sugars, trigonelline and chlorogenic acid are formed in unwanted substances, such as furfural-type compounds, including 5-hydroxymethylfurfural (5-HMF), 2-acetylfuran (FMC) and 5-methyl-2-furfural (MF) [7]. These compounds are found in foods affected by storage or processing, or that have undergone heat treatment or enzymatic browning reactions [8]. 5-HMF is an intermediate compound formed by enzymatic browning or Maillard reactions, formed by the dehydration of fructose and glucose in an acidic medium [9]. HMF with the IUPAC name of 5-(hydroxymethyl) furan-2-carbaldehyde has a molecular weight of 126.11 g mol^−1^ and consists of a furan ring with functional groups, such as aldehyde or alcohol. HMF concentrations in roasted coffee have been found between 300 and 2900 mg kg^−1^ [10]. Furans and methylfurans are VOCs found in a wide variety of foods when they undergo thermal processing, and they present adverse health effects [11]. This compound is a cyclic aldehyde that is absent in fresh foods, but when it appears in foods through the aforementioned reactions, it is considered potentially carcinogenic to humans. Analyzing the number of toxic substances in foods is therefore essential [12], and many countries have established policies to control their content in foods. However, the direct quantification of these compounds continues to pose a challenge due mainly to the complexity of the samples and interfering substances [13].

A further undesirable compound formed during the coffee roasting process is acrylamide—a compound classed as carcinogenic by the Food Safety and Nutrition Agency [14]. This compound is included as a toxic, carcinogenic compound classified in group 2A [14] with a reference level of acrylamide between 50 and 100 μg kg^−1^ [15,16]. Asparagine is the main amino acid precursor that is converted during the Maillard reactions after undergoing a decarboxylation and deamination reaction in acrylamide [17]. Therefore, this product represents one of the principal sources of acrylamide with high daily consumption [18].

The precursor compounds of coffee aroma include lipids, which undergo autooxidation and decomposition reactions during the roasting process, contributing to the formation of an aroma in coffee-type beverages [19].

The concentration of these toxic compounds is quantified using gas and liquid chromatography. Gas chromatography technology is used for the quantification of VOCs, while liquid chromatography is used to evaluate the amount of hydroxymethylfurfural [10,20]. Organoleptic techniques are also useful to evaluate the final product quality [20,21,22]. This requires specialized people trained in obtaining high-quality results. In addition, such sensory evaluations require appropriate facilities, the preparation of samples and the organization of tasting sessions [23]. The aromatic profile of roasted coffee can, however, also be characterized using sensor techniques, such as an electronic nose (e-nose)—a promising tool with great potential for analysis [24]. It is a non-destructive, low-cost, efficient and fast technique [25] that has been used by different researchers [26,27] to analyze volatile compounds in foods and food defects. Its sensors react with specific aromatic molecules that produce a signal that generates a fingerprint of the sample to be evaluated. The data obtained are transformed into digital data and processed by algorithms and machine learning techniques to represent the results by applying different multivariate analyses [23,25,26]. Therefore, coffee beverages were evaluated whose beans had been submitted to different thermal treatments by analyzing the sensory profile, content of toxic compounds, volatile organic compounds and e-nose data. The aim was to establish relationships between this data and the aromas to indirectly evaluate the content of acrylamide and 5-hydroxymethylfurfural.

## 2. Materials and Methods

### 2.1. Samples

**Figure 1:**
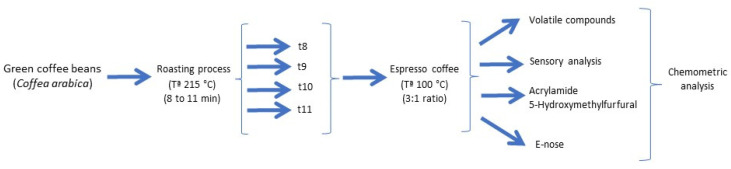
Diagram of experiment.

Two kg of green Arabica coffee (*Coffea arabica* L.) samples were harvested in the Copán region (Honduras) during the 2022/23 crop season. Coffee from Honduras was selected as it is a coffee that competes in quality with other varieties. Furthermore, it is an emerging market in terms of organic coffee, and due to its organoleptic properties, like Colombian coffee, Honduran coffee is a quality Arabica coffee. Fresh samples of 150 g were placed onto porcelain plates to be roasted in a conventional oven (model 210, J.P. Selecta^®^, Barcelona, Spain) at 215 °C, as described by Barea-Ramos et al. [28]. The roasting times were 8, 9, 10 and 11 min, respectively. Samples were roasted in triplicate. The roasted samples were stored at room temperature for 48 h, after which a coffee beverage was made. The samples were mechanically ground at a constant speed for 1 min. The ground coffee size measured 0.4 mm. Next, an espresso coffee beverage was prepared by adding hot mineral water (100 °C) to roasted coffee at a ratio of 3:1, respectively. The espresso was brewed at 9 bar pressure. Samples of 30 mL each were prepared in triplicate. The different brewed beverages were analyzed via a sensory panel, gas chromatography and an e-nose. Figure 1 depicts the study carried out.

### 2.2. Analysis of Volatile Compounds

The VOCs of samples were evaluated using gas chromatography–mass equipment to determine the profile of their aromatic compounds, as per the method described by Sánchez et al. [29]. Samples of 2 g of each beverage were placed into a vial, and the aroma was absorbed using polydimethylsiloxane/divinylbenzene (PDMS/DVB) StableFlex fiber (65 μm, Supelco). A triple quadrupole mass spectrometry detector model 456-GC was applied. The fiber was injected into this equipment. A capillary column Agilent DB WAXetr (60 m × 0.25 mm; DI: 0.25 mm) was used. The desorption was carried out at 250 °C for 15 min. Each peak of the samples studied was identified using the NIST 2.0 MS library. VOCs were measured in %.

### 2.3. Sensory Analysis

The aroma characteristics of the espresso coffee were assessed by a tasting panel [23] made up of eight experts in food sensory evaluation at the University of Extremadura. The evaluation test was carried out in a tasting room. Samples were prepared in a tasting glass and evaluated immediately. The positive attribute evaluated was coffee aroma, while the negative attribute was that of a roasted/burnt odor. The results of each taster were included in a structured scale (0–10 points) and were evaluated by the head of the tasting panel when the coefficient of variation was less than 20. The median of the evaluated results was made for each of the attributes studied.

### 2.4. Acrylamide Analysis

The protocol analysis described by Pérez-Nevado et al. [30] was used to calculate the acrylamide content in the coffee beverage studied. A quantity of 2 g of coffee was filtered through a 0.45 μm nylon syringe filter, and the liquid was placed into two Telos columns (PCX, 200 mg/3 mL and PRP, 60 mg/3 mL) to obtain the eluate. Concentrations ranged from 50 to 150 ng mL^−1^ of acrylamide, and a standard addition method was used. Finally, an Agilent 1290 Infinity II liquid chromatograph (Agilent Technologies) coupled to an Agilent 6460 triple quadrupole mass spectrometer was used to obtain the concentration of this toxic substance. Helium was used as carrier gas with a flow rate of 0.8 mL min^−1^. It was worked in isocratic mode with a flow of 0.25 mL min^−1^, using eluent A (0.1% formic acid in Milli-Q-water) and eluent B (0.1% formic acid in methanol). The gas injector temperature was 340 °C, the nebulizer pressure was 40 psi, the sheath gas temperature was 400 °C with a flow rate of 12 L h^−1^ and capillary voltages were +2.5 kV. The nozzle voltage was 300 V and dental EMV: 300.

### 2.5. 5-Hydroxymethylfurfural Analysis

The quantification of 5-HMF was carried out as per the methodology proposed by Long et al. [31]. A Shimadzu HPLC system (Tokyo, Japan) was coupled to a Thermo Q-Exactive Plus mass spectrometer (USA). A C18 column (2.1 mm × 100 mm × 1.9 mm) (Waters Co., Torrance, CA, USA) was used. The mobile phases used were acetonitrile (eluent B) and formic acid at 0.1% (*v*/*v*) in water (eluent A) with a flow rate of 0.3 mL min^−1^ and an injection volume of 2 μL and were worked into an elution system in gradient as follows: Elution of 100% of eluent A up to 1.20 min. Furthermore, 80% of eluent A for 1.21–1.80 min. Subsequently, 80% of eluent B and 40% of eluent A for 1.81–3.0 min and 40%–10% of A for 3.01–4.50 min, which was the time required for the elution of each sample. The amounts of 5-HMF were quantified by external calibration using 5-HMF reference standards expressed in μg L^−1^. A UV–visible detector at a wavelength of 280 nm was used. The peaks obtained were compared with the retention times of the standards and quantified by calculating the peak area corresponding to the HFM in the chromatograms.

### 2.6. E-Nose Measurements

The samples were additionally analyzed in the laboratory using an e-nose equipment prototype designed by the Engineering Department of Miguel Hernández University (Spain) in collaboration with Telenatura EBT, S.L. (Elche, Spain) [32]. The e-nose comprises a chamber for depositing samples, an air pump or fan, a sensor matrix and a data-processing unit (Arduino Nano microcontroller with USB serial connection). The sensor array consists of eight MQS sensors manufactured by Hanwei Electronics Co., Ltd., Zhengzhou, China. These metal oxide semiconductor resistive sensors detect changes in electrical resistance when exposed to specific gases, offering a wide-ranging detection of VOCs. The MQ resistive sensors were as follows: MQ2 (liquefied petroleum gases (LPG), hydrogen and propane), MQ3 (alcohol), MQ4 (methane), MQ5 (hydrogen and LPG), MQ7 (hydrogen and carbon monoxide), MQ8 (hydrogen), MQ9 (carbon monoxide and LPG), MQ135 (NH_3_ (ammonia), NO_x_, alcohol, benzene, smoke, CO_2_, etc.). The device included an Arduino Nano microcontroller and an analog circuit for controlling sensor heating with sensor sensitivity enhanced through voltage modulation. A sinusoidal voltage variation strategy was employed with a period of 128 s and a voltage range from 1.6 to 4.8 V. Normalization techniques, including the use of 50 kΩ trimmer potentiometers as load resistors, were applied to balance sensor outputs and account for manufacturing variability. A calibration process preceded analysis, defining parameters, such as stabilization time (SBT), sensing time (ST), cleaning time (CT), total time (OVT) and maximum number of analyses (MNA), ensuring experiment completion within five hours to maintain test result integrity.

The experiments were conducted in a clean and disinfected environment. A sample of 10 mL of freshly brewed coffee was placed into a chamber. Next, the chamber was hermetically closed, and the quantum light sensor (PAR) Walz (MQS) was used to evaluate the aroma of each sample. Prior to placing the next samples, the chamber was cleaned, and the sensors were exposed to clean air for 15 min. Data were extracted using computer software and a chemometric analysis was carried out.

### 2.7. Multivariate Data Analysis

The raw e-nose data were processed by applying an algorithm. Thus, each group of data obtained by this equipment was recalculated by applying the maximum signal value minus the minimum signal data, multiplied by 100 and subtracted by one. These data were used to differentiate between the different treatments studied by applying principal components analysis [20,23]. Furthermore, the evaluation of coffee beverage aromas perceived by the panelists and the negative aromas were related by applying a partial least squares regression test [30]. PLS_Toolbox 8.2.1 (Eigenvector Research Inc., Wenatchee, WA, USA) associated with Matlab version R2016b, version 9.1 (The Mathworks Inc., Natick, MA, USA) was used to analyze the data.

### 2.8. Statistical Analysis

An analysis of variance was carried out to establish differences between the roasted coffee beverages studied. The differences between each thermal treatment were discriminated by Tukey’s test. The statistical program used was SPSS 18.0 (SPSS Inc., Chicago, IL, USA). Furthermore, the relation between the aroma perception and the toxic substance was established using multiple linear regression. Finally, the coefficient of determination (R^2^) was indicated in each model established.

## 3. Results and Discussion

### 3.1. Effect of Roasted Coffee on Volatile Organic Compounds

**Figure 2:**
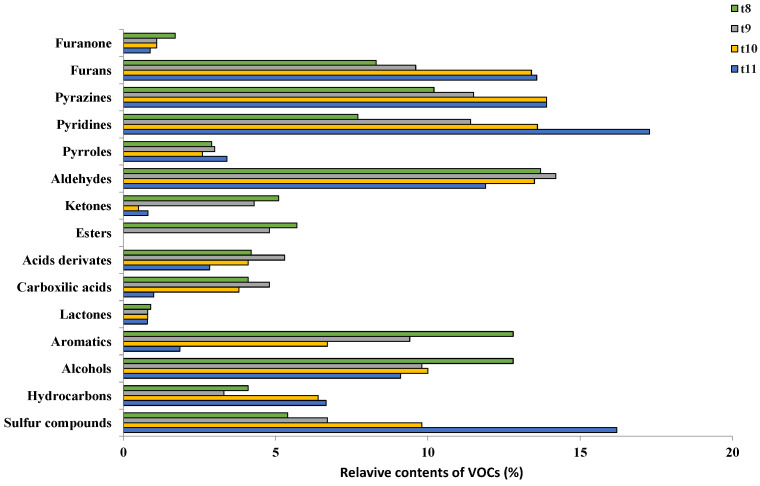
Chemical distribution of volatile compounds (%) in roasted coffee beverage.

Figure 2 shows the percentage of the different VOCs formed in the beverage at different roasting times (8, 9, 10 and 11 min). They were grouped into 15 chemical families, and those presenting the highest percentage of VOCs were furans, pyrazines, pyridines and aldehydes. The minor ones were pyrroles, furanones and lactones.

The concentration of pyridines was seen to increase with the roasting time up to 36.3%. Pyridines, as well as other types of compounds that contain heterocyclic rings, such as pyrazines, pyrroles or imidazoles, contribute to Maillard reactions with the production of melanoidins. This fact is related to the increase in Maillard reactions with longer roasting times [14]. Furans also increased, especially after 9 min. The formation of furans is related to the sweet caramel flavor and burnt smell of coffee [33]. Aldehydes together with organic acids, alcohols and esters are the families of compounds crucial as precursors of the VOCs responsible for aroma in the coffee roasting process [34]. The percentage of aromatic compounds and alcohols was the same with a roasting time of 8 min (12.8%). The aromatic compounds decreased over time, by up to 85.5% for the roasting time of 11 min. These types of compounds are essential in the production of coffee aroma both in the drying and roasting phases [23,28].

Ketones presented 5.1% of VOCs for a roasting time of 8 min and decreased by 90.2% at 10 min. Some esters were also present for the roasting times of 8 and 9 min but then disappeared when the time increased. Carboxylic acid derivatives increased by 26.2% when the roasting time increased from 8 to 9 min but decreased by 67.6% when it reached 11 min. Carboxylic acids and their derivatives presented the same pattern. These increased for the roasting time of 9 min and then decreased by up to 64.8% for the roasting time of 11 min in relation to the acid derivatives. The increase in carboxylic acids and derivatives when the roasting time increased could be attributed to the degradation of carbohydrates and esters that subsequently degrade if the roasting time continues to increase [35]. Hydrocarbon compounds, specifically polycyclic hydrocarbon compounds, increased by 62.4% during the roasting process. These are undesirable compounds formed during the roasting process and are considered genotoxic and carcinogenic to humans [36]. Sulfur compounds are also formed in the roasting process. The concentration of these compounds increased the most, reaching up to 264% for a roasting time of 11 min. The sulfur family is an essential class with low positive aromatic thresholds. Sulfides that include compounds such as thiols, thiophenes and thiazoles are formed by Maillard reactions from cysteine and present a toasted and garlicky aroma [37].

Finally, among the minor VOCs found during coffee roasting were pyrroles—compounds formed by an aromatic ring of four carbon atoms and a nitrogen atom whose concentration increased in coffee with the degree of roasting [38]. These presented no differences in the first two roasting times and subsequently increased when the coffee was roasted up to 11 min. These compounds are formed through the degradation of Strecker in the coffee roasting process, negatively affecting its sensory quality [39]. Another group of minor VOCs found were furanone-type carbohydrates, which, although their formation was only 1.7% for a roasting time of 8 min, reduced by 53.0% for a roasting time of 11 min. Carbohydrates, along with chlorogenic acids, are degraded into other compounds that are formed such as furans, pyrazines and lactones [40]. Although lactones appeared, there were no significant differences in this type of substance during the different roasting times.

A total of 70 VOCs were identified for the four roasting times and are presented in Table 1. They were classified into 15 chemical families grouped as follows: three furanones, four furans, six pyrazines, four pyridines, four pyrroles, six aldehydes, five ketones, five esters, four acid derivatives, four carboxylic acids, two lactones, six aromatics, eight alcohols, six hydrocarbons and three sulfur compounds. Of these, the unwanted compounds that were formed in greater concentrations with the increase in roasting time and consequently contributed negatively to the quality of the coffee were furans. Their main compounds were furfural and 2-methylfurfural, the latter presenting concentrations 2.23 times higher than in furfural for the roasting time of 11 min. These types of compounds that are formed during the thermal processes of coffee roasting are classified as carcinogenic to humans since they are transformed into 2-sulfoxymethulfuran and 5-sulfoxymethylfurfural that can create mutations by reacting with DNA or proteins [41]. Pyrazines represent another family of compounds whose formation in the coffee roasting process contributes negatively to the quality of coffee. Among these types of compounds, 2-ethylpyrizine and 2-ethyl-6-methylpyrizine stood out. The concentration for both these compounds doubled with increasing roasting times. The formation of these types of compounds in the roasting process is related to the pyrolysis of hydroxyamino acids, trigonelline and sugars [42]. Pyrazines, along with pyrroles and pyridines, are responsible for contributing roasted and nutty attributes in coffee [43]. Among the pyridines found in the coffee roasting process were pyridine and 2,5-dimethyl-pyridine. The concentration of pyridine increased threefold when the roasting time was increased from 8 to 11 min, and for 2,5-dimethyl-pyridine, the concentration increased fourfold in relation to the roasting time of 8 min. Of the five pyrroles identified, some were degraded during the roasting process, except for 1H-Indole, whose concentration increased by 250%. Pyrroles also give coffee certain buttery notes similar to caramel [23].

The concentrations of carbonyl compounds presented significant differences with the coffee roasting time. Ketones were degraded as the time increased, and among the aldehydes that contributed negatively to the quality of the coffee was 2-methyl-butanal. Its concentration tripled for the roasting time of 11 min compared with that of 8 min, with hexanal being present at the beginning of the roasting process and then reduced by half over the roasting time. Both compounds are crucially important to the quality of coffee, especially 2-methyl-butanal, which gives malty notes, and hexanal, which is formed during coffee storage and can contribute to the rancid flavor due to lipid oxidation [44]. The concentration of carboxylic acids and derivatives generally decreased until they were completely degraded during the roasting time. Few of these compounds were still present at the 11th minute. Those that stood out were nonanoic acids, whose concentration at the roasting time of 11 min was reduced to a third compared with the roasting time of 8 min, and carboxylic acid, namely 3-methyl-butanoic acid [45], whose concentration was maintained without significant differences during the coffee roasting process.

The lactones detected in the different coffee treatments presented no significant differences with the roasting time. Some lactones are formed in the roasting process as chlorogenic acids which give coffee a certain bitterness [46]. Alcohols also degraded over time, except for 1-butanal-3-methyl, whose concentration increased slightly. Of the six hydrocarbons detected, all showed low concentrations, and there were no significant differences between the roasting times with the exception of tetradecane, whose concentration at 11 min was four times higher than at 8 min. Finally, other families linked to the negative quality of coffee were sulfide compounds, namely dimethyl sulfide, 2-furfurylthiol and 2-furanmethenethiol. The concentration of the former reduced slightly with the increase in roasting time from 8 to 9 min before stabilizing. 2-furfurylthiol increased by 78.8% for the roasting time of 11 min compared with the roasting time of 8 min, and 2-furanmethenethiol also increased by 69.0% compared with the roasting time of 8 min. These thiols lend freshly roasted coffee a characteristic aroma. Unfortunately, this compound reduces rapidly during storage as the result of polymerization or oxidation reactions [47].

### 3.2. Effect of Roasted Coffee on Sensory Analysis

The roasted coffee beverage underwent a sensory analysis by panelists to describe the positive and negative aroma (Table 2). The espresso coffee made with fresh coffee beans submitted to low thermal treatments presented a good aroma, and the tasters detected no negative attribute. Thus, the t8 treatment could be the thermal treatment that presented the highest quality as no defect was detected. The strongest coffee aroma was observed in the t9 treatment, but some negative defects were also detected. Moreover, when the heat intensity in the fresh coffee beans increased (t9 to t11), negative defects appeared. The roasted aroma is an attribute related to a burnt defect that tasters detected when high intensity thermal treatment was applied. This roasted smell is related to the undesirable compounds that are formed as the coffee roasting time is increased, as described in Table 1.

The results of the roasted coffee beverage demonstrate that the analysis carried out by panelists was able to classify the samples evaluated. Stokes et al. [7] indicated this odor as a negative attribute in fresh filter coffee. As the fresh coffee was processed in the same way, it is clearly the intensity of the heat treatment that caused an increase in the sensory defect, in turn decreasing the characteristic aroma of the coffee beverage. Chapko and Seo [48] evaluated the sensory attributes of brewed coffee submitted to different serving temperatures indicating that the highest temperatures studied implied high coffee aromas with some negative attributes. This result showed us the importance of applying the appropriate thermal treatment to obtain the highest quality of brewed coffee. Thus, consumers will appreciate roasted coffee that provides the best sensory characteristics to the final product.

### 3.3. Effect of Roasted Coffee on Chemical Contaminants

The acrylamide and 5-hydroxymethylfurfural levels of the roasted coffee beverage submitted to different thermal treatments are shown in Table 3. Thermal treatments applied to fresh coffee beans had a significant effect on the production of these toxic chemicals in the roasted coffee beverage. The highest amount of acrylamide was observed in the softest thermal treatment. The amount of this substance then decreased progressively until values of the highest thermal treatment measured less than 60%. However, the 5-hydroxymethylfurfural concentration increased when the thermal treatment was more aggressive. An almost fourfold increase in this toxic substance was found when the thermal treatment was higher. The acrylamide content was higher than the concentration of 5-hydroxymethylfurfural in all the elaborated coffee beverages.

**Figure 3:**
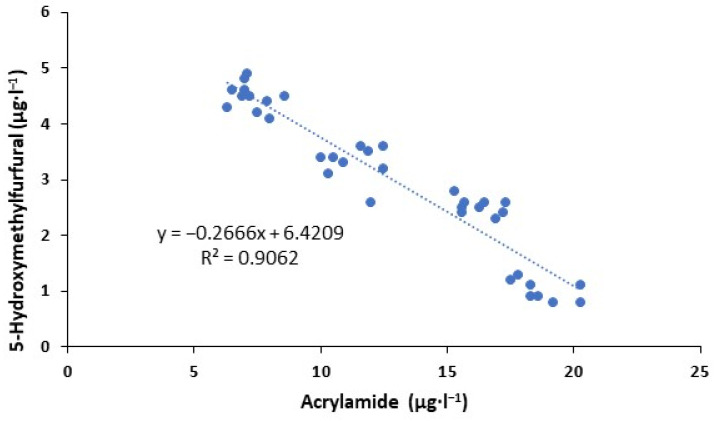
Relationship between acrylamide and 5-hydroxymethylfurfural content in roasted coffee beverage.

A negative correlation (R^2^ = 0.91) was found between the acrylamide concentration and 5-hydroxymethylfufural (Figure 3) when considering the high influence of thermal treatments in the formation of these substances. When the concentration of acrylamide in the roasted coffee beverage increased, the 5-hydroxymethylfurfural content was lower, while a lower concentration of acrylamide led to a beverage with a higher 5-hydroxymethylfurfural content. This inverse linear relationship was also observed by Lachenmeier et al. [49].

Heat treatment is a necessary step in the coffee making process, but despite applying low thermal treatments, harmful toxic substances are also produced. Concentrations of acrylamide increased in the lowest intensity of the thermal treatment. This produces a wide range of undesirable substances in the final product that depends on the intensity of the thermal treatment applied. Different researchers [49,50] indicated that different brewed coffee preparations produced high levels of acrylamide content. Furthermore, when the heat applied to fresh coffee was too high, the level of acrylamide content decreased significantly [51]. However, 5-hydroxymethylfurfural contents gradually increased with the increasing intensity of the applied heat treatments. Mesías et al. [50] highlighted the content of 5-hydroxymethylfurfural in brewed coffee of the Arabica and Robusta variety. The amount of this toxic substance increased with increasing the roasting temperature/time of the elaboration process [52].

We need to highlight that ingesting two coffees per day would cause us to ingest an average of 0.56 μg and 0.162 μg of acrylamide and 5-hydroxymethylfurfural, respectively. It is estimated that the population could ingest 0.037 μg of acrylamide per kg of body weight a day and 0.021 μg of 5-hydroxymethylfurfural per person per day. Thus, for a person weighing 70 kg, the maximum dose of ingestion a day of these toxic substances rises to 2.59 μg and 1.47 μg of acrylamide and 5-hydroxymethylfurfural, respectively [50,53]. Therefore, it is suggested that the ingestion of a low number of roasted coffee beverages does not imply a high risk in the population and more specifically in the adult population. Consumption should be moderated in elderly people and adolescents, especially when other foods likely to contain these toxic substances produced in the manufacturing process are ingested.

### 3.4. Relationship between Chemical Contaminants and Perceived Sensory Defect

**Figure 4:**
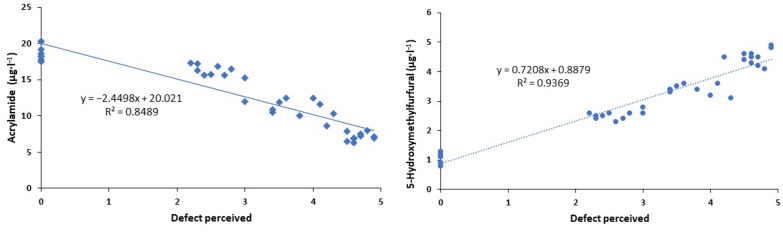
Relationship between toxic compounds and perceived sensory defect.

As observed in the previous section, the toxic compounds detected in the roasted coffee beverage varied depending on the intensity of the treatments applied to the fresh coffee beans. Therefore, a linear regression was carried out between the toxic compounds and the sensory defect detected by the tasting panel (Figure 4). As can be seen, an increase in the roasted or burnt defect implied a decrease in the acrylamide content (R^2^ = 0.85) and an increase in the 5-hydroxymethylfurfural concentration (R^2^ = 0.94). Fresh coffee beans subjected to different treatments caused a sensory defect in the brewed beverage. This sensory variable had a good correlation between the toxic compounds studied. This would allow us to predict the content of toxic compounds by carrying out an organoleptic assessment of the final product. Researchers [54,55] have found relationships between toxic substances and sensory parameters in different products in an attempt to predict the content of these compounds.

### 3.5. E-Nose Capacity to Discriminate Roasted Coffee Beverage

**Figure 5:**
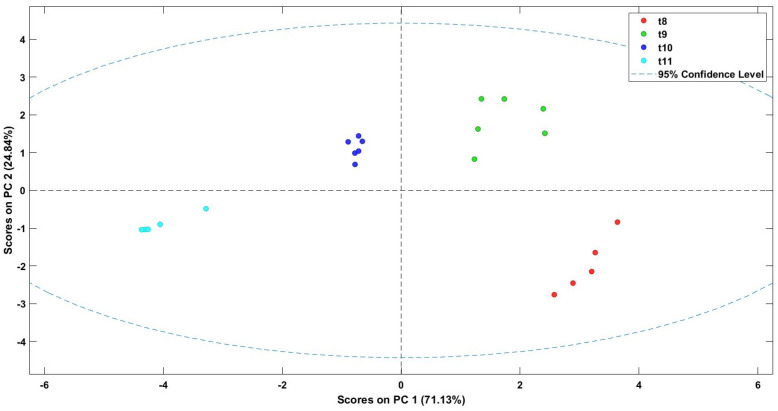
Score plot of PCA in roasted coffee beverage.

The aromas of the roasted coffee beverage were also studied using an electronic device to discriminate them based on their volatile organic compounds. Principal component analysis showed that this device discriminated the samples according to their aroma (Figure 5). The variance was described by PC1 at 71.1%, while PC2 explained the 24.8%. As can be seen in the figure, the different roasted treatments applied to coffee beans provoked different aromas in the final product. The sensors of the electronic device reacted in different ways according to the volatile organic compounds detected. This low-cost electronic device could be useful for consumers since it could allow for a preliminary classification of the aroma of brewed roasted coffee after applying different thermal treatments to fresh coffee beans. The e-nose has been used to create odor fingerprints of different varieties of roasted coffee [56] or even to obtain a sensory profile of different drying methods [57]. It has also been used to discriminate the aromatic profile of volatile compounds in the roasting process of other species, such as *Pinus koraiensis* [58].

**Figure 6:**
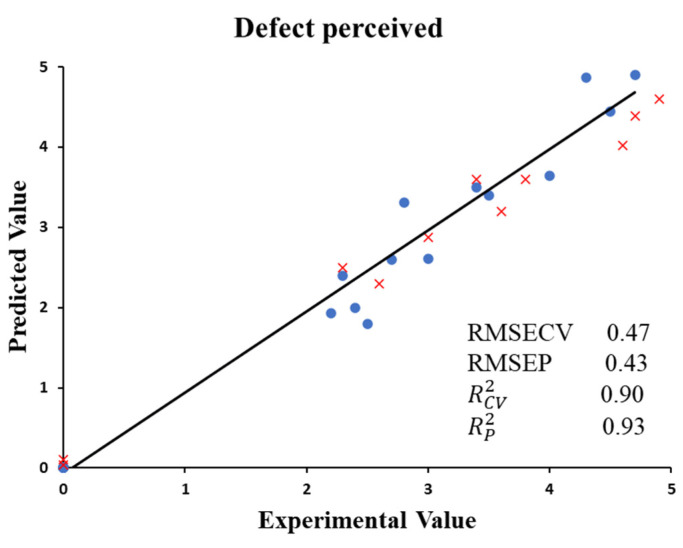
PLS cross-validation predictions (●) and validation set predictions (x) for defect aroma perceived and e-nose data.

Finally, a PLS model was carried out between the data obtained with the electronic device and the sensory defect perceived by the tasters (Figure 6). The figure shows a linear prediction model between the negative aroma perceived and the data obtained by the electronic sensors’ signals. $R_{CV}^{2}$ values for the defect perceived were 0.90. A low RMSECV value of 0.47 was also assessed. The model was validated using external samples. $R_{P}^{2}$ values were 0.93 for negative aromas perceived, whilst RMSEP values were 0.43. Thus, this model led to the quantification of the defect detected by the panelists of the roasted coffee beverage samples. The scientific literature contains studies that discriminate coffee samples with electronic devices. Some researchers [28] discriminated coffee samples of different qualities from Colombia. Others [59] classified coffee submitted to different intensities of roasting periods. Zhang et al. [60] differentiated Robusta coffee using an e-nose during the application of different heat air-drying treatments. Furthermore, the evolution of chemical aromatic compounds in espresso coffee was evaluated using an e-nose system.

Therefore, via an indirect measurement, it would be possible to quantify the number of toxic compounds in the roasted coffee beverage using an electronic device since there is a correlation between the sensory defect and toxic compounds as well as between the e-nose and the defective roasted aroma of coffee.

## 4. Conclusions

When fresh coffee beans are subjected to different intensities of heat treatment, different intensities of positive and negative aromas are produced in the brewed coffee beverage. All of these aromas are characterized by different VOC profiles. The toasted or burnt defect caused by excessive roasting of the beans results in a positive or negative aroma that is easily detected by specialized tasters. The application of heat to coffee beans produces the synthesis of toxic compounds in different concentrations in roasted espresso coffee, specifically acrylamide and 5-hydroxymethylfurfural. The amount of these compounds is related to the aromatic profile of the coffee samples. Significant relationships were observed between these toxic compounds and acrylamide levels, since elevated acrylamide levels corresponded to decreased amounts of 5-hydroxymethylfurfural. The e-nose was able to detect the olfactory pattern of the roasted samples, establishing a linear classification between the levels of toxic substances and the sensory defect. A PLS model showed a relationship between the defect perceived by the tasters and the response of the electronic sensors, since a high sensory defect indicated a decrease in acrylamide and high content of 5-hydroxymethylfurfural. Furthermore, the electronic signals exhibited a correlation with the sensory defect, thus allowing us to anticipate the presence of these contaminants in roasted coffee beverages. Therefore, this device could be considered useful as an indirect predictive methodology to discriminate the aroma of a coffee beverage and could indirectly identify the number of toxic compounds in the brewed espresso by virtue of its sensory attributes.

## Figures and Tables

**Table 1:** Content of volatile compounds (mean %, triplicate) obtained from roasted coffee beverage.

	**Furanone**			**Furans**				**Pyrazines**							
**Treatments (min)**	**Dihydro-5-methyl-2(3H)-furanone**	**Dihydro-2-methyl-2(3H)-furanone**	**2(5H)-Furanone**	**Furfural**	**2-Methyl-furan**	**5-Methyl-furan**	**Furan**	**Pyrazines**	**2-Methyl-pyrazine**	**2-Ethylpyrazine**	**2,3-Dimethylpyrazine**	**2,3,5-Trimethylpyrazine**	**2-Ethyl-6-methylpyrazine**		
t8	0.5	0.5	0.7	1.5	2.5	2.0	2.3	1.6	1.5	2.0	2.0	1.5	1.6		
t9	0.5	0.0	0.6	2.1	2.4	2.6	2.5	1.5	1.5	1.9	2.4	1.6	2.6		
t10	0.6	0.0	0.5	2.6	5.5	3.0	2.3	2.0	2.2	3.5	2.0	1.4	2.8		
t11	0.3	0.0	0.6	3.5	7.8	0.0	2.3	1.5	2.5	3.6	1.6	1.5	3.2		
	**Pyridines**					**Pyrroles**				**Aldehydes**					
**Treatments (min)**	**Pyridine**	**2-Methoxypyridine**	**3-Ethylpyridine**	**Pyridine, 4-ethenyl-**	**2,5-Dimethyl-pyridine**	**1-Methyl pyrrole**	**1-Furfuryl pyrrole**	**1H-Pyrrole**	**1H-Indole**	**Butanal**	**2-Butenal**	**2-Methyl-butanal**	**Hexanal**	**Benzaldehyde**	**Nonanal**
t8	2.5	0.6	1.5	0.5	2.6	0.6	0.5	1.2	0.6	1.2	0.5	2.3	2.0	0.5	7.2
t9	5.4	0.5	1.2	0.5	3.8	0.5	0.5	1.0	1.0	1.0	0.6	3.0	2.8	0.8	6.0
t10	6.4	0.6	1.1	0.0	5.5	0.6	0.4	0.0	1.6	0.0	0.5	5.5	1.6	0.7	5.2
t11	7.0	0.5	0.0	0.0	9.8	0.7	0.6	0.0	2.1	0.0	0.5	7.2	1.2	0.8	2.2
	**Ketones**					**Esters**					**Acids derivates**				
**Treatments (min)**	**2-Nonanone**	**2-Heptanone**	**Propenone, 1-(4-nitrophenyl)-3-phenylamino**	**Geraniol**	**2-Pentadecanone, 6,10,14-trimethyl**	**Ethyl acetate**	**2-Propenoic acid, butyl ester**	**Acetic acid, 2-ethylhexyl ester**	**2-Methyl-propanoic acid, octyl ester**	**Hexadecanoic acid, ethyl ester**	**3-Methyl-butanoic acid**	**Hexanoic acid, 2-methyl**	**2-Methyl-butanoic acid**	**3-Methyl-2-butenoic acid**	
t8	0.5	0.5	2.6	1.0	0.5	0.6	0.4	1.5	2.2	1.0	2.0	1.0	0.0	1.2	
t9	0.6	0.6	1.5	1.2	0.4	0.6	0.5	1.2	1.5	1.0	2.4	0.4	1.5	1.0	
t10	0.5	0.0	0.0	0.0	0.0	0.0	0.0	0.0	0.0	0.0	2.5	0.4	0.0	1.2	
t11	0.0	0.0	0.0	0.8	0.0	0.0	0.0	0.0	0.0	0.0	2.8	0.0	0.0	0.0	
	**Carboxylic acids**				**Lactones**		**Aromatics**								
**Treatments (min)**	**Acetic acid**	**Dentanoic acid**	**Hexanoic acid**	**Nonanoic acid**	**γ-Butyrolactone**	**2,3-Pentanedione**	**Phenol**	**3-Methyl-phenol**	**D-Limonene o 1-Metil-4-(1-metiletenil)-ciclohexeno**	**2-methoxy-phenol (Guaiacol)**	**2-Methoxy-4-vinylphenol**	**Benzene, 2,4-diisocyanato-1-methyl-**			
t8	0.0	0.5	0.6	3.0	0.4	0.5	2.5	0.8	2.0	0.5	5.0	2.0			
t9	1.0	0.4	0.4	3.0	0.4	0.4	2.6	0.8	2.4	0.6	2.0	1.0			
t10	1.0	0.5	1.1	1.2	0.4	0.4	2.0	0.7	2.0	0.0	2.0	0.0			
t11	0.0	0.0	0.0	1.0	0.4	0.4	0.0	0.5	1.4	0.0	0.0	0.0			
	**Alcohols**								**Hydrocarbons**						
**Treatments (min)**	**1-Butanol, 3-methyl-**	**1-Pentanol**	**2,3-Butanediol**	**1-Hexanol**	**1-Octen-3-ol**	**1-Propanol**	**Benzyl alcohol**	**2-Phenylethyl alcohol**	**1,6-Octadien-3-ol, 3,7-dimethyl- (Linalool)**	**Toluene**	**Pentane**	**Styrene**	**Pentadecane**	**Tetradecane**	
t8	1.6	0.5	0.0	2.5	1.0	0.8	2.0	4.4	0.7	0.7	0.5	0.6	0.5	1.1	
t9	1.6	0.0	0.6	2.0	1.0	1.2	1.3	2.1	0.4	0.5	0.4	0.5	0.5	1.0	
t10	2.0	0.0	0.5	1.2	1.0	1.1	1.0	3.2	0.4	0.5	0.6	0.6	0.6	3.7	
t11	2.3	0.0	0.9	0.6	1.2	1.3	0.5	2.2	0.4	0.5	0.5	0.5	0.4	4.4	
	**Sulfur compounds**														
**Treatments (min)**	**Dimethyl sulfide**	**2-Furfurylthiol**	**2-Furanmethanethiol**												
t8	1.5	1.7	2.2												
t9	1.0	2.5	3.2												
t10	1.0	3.8	5.0												
t11	1.1	8.0	7.1												

**Table 2:** Sensory assessment of roasted coffee beverage (mean ± standard deviation). Small letters indicate significant statistical differences between the different experimental coffee elaborated (Tukey’s test, *p* < 0.05).

t (min)	Aroma	
	Coffee	Roasted/Burnt
t8	4.2 ± 0.2 b	n.d.
t9	6.3 ± 0.2 a	2.5 ± 0.2 c
t10	3.6 ± 0.2 c	3.5 ± 0.3 b
t11	2.8 ± 0.3 d	4.7 ± 0.5 a

n.d.: not detected.

**Table 3:** Acrylamide and 5-hydroxymethylfurfural in roasted coffee beverage (mean ± standard deviation). Small letters indicate significant statistical differences between the coffee elaborated after applying different thermal treatments to coffee beans (Tukey’s test, *p* < 0.05).

t (min)	Acrylamide (µg∙L^−1^)	5-Hydroxymethylfurfural (µg∙L^−1^)
t8	18.5 ± 0.7 a	1.1 ± 0.1 d
t9	16.1 ± 1.1 b	2.6 ± 0.2 c
t10	11.3 ± 0.6 c	3.4 ± 0.2 b
t11	7.3 ± 0.3 d	4.5 ± 0.1 a

## Data Availability

The original contributions presented in the study are included in the article, further inquiries can be directed to the corresponding author.

## References

[1] Othman N., Muhammad E. (2019). Drying of instant coffee in a spray dryer. J. Kejuruter..

[2] Lee C.H., Rianto B. (2024). An Al-powered e-nose system using a density-based clustering methods for identifying adulteration in specialty coffees. Microchem. J..

[3] Wintgens J.N. (2004). Coffee: Growing, processing, sustainable production. Botany and Genetics of Coffee.

[4] Sunarharum W.B., Williams D.J., Smyth E. (2014). Complexity of coffee flavor: A compositional and sensory perspective. Food Res. Int..

[5] Bona E., da Silva R.S.S. (2016). Coffee and the Electronic Nose. Electronic Noses and Tongues in Food Science.

[6] Owczarek-Fendor A., de Meulanaer B., Scholl G., Adams A., Van Lancker F., Eppe G., De Pauw E., Scippo M.L., de Kimpe N. (2011). Furan formation from lipids in starch-based model systems, as influenced by interactions with antioxidants and proteins. J. Agric. Food Chem..

[7] Stokes C.N., O’Sullivan M.G., Kerry J.P. (2017). Hedonic and descriptive sensory evaluation of instant and fresh coffee products European. Food Res. Technol..

[8] Gómez-Narváez F., Pérez-Martínez L., Contreras-Calderón J. (2019). Usefulness of some Maillard reaction indicators for monitoring the heat damage of whey powder under conditions applicable to spray drying. Int. Dairy J..

[9] Kowalski S., Lukasiewicz M., Duda-Chodak A., Ziec G. (2013). 5-Hydroxymethyl-2- Furfural (HMF)-heat-induced formation, occurrence in food and biotransformation—A Review. Pol. J. Food Nutr. Sci..

[10] Murkovic M., Pichler N. (2006). Analysis of 5-hydroxymethylfurfual in coffee dried fruits and urine. Mol. Nutr. Food Res..

[11] Rahn A., Yeretzian C. (2019). Impact of consumer behavior on furan and furan-derivative exposure during coffee consumption. A comparison between brewing methods and drinking preferences. Food Chem..

[12] Didaba T., Tilahun L., Satheesh N., Geremu M. (2018). Acrylamide occurrence in Keribo: Ethiopian traditional fermented beverage. Food Control.

[13] Gan Y.M., Li K.X., Zhang N., Xu X., Chen D. (2023). Current sample preparation strategies for the chromatographic and mass spectrometric determination of furfural compounds. Microchem. J..

[14] IARC (2012). Coke Production. A review of human carcinogens: Chemical agents and related occupations. IARC Monographs on the Evaluation of the Carcinogenic Risk of Chemicals to Humans.

[15] Mayerhofer U., Czerwenka C., Marchart K., Steinwider J., Hofstaedter D. (2019). Dietary exposure to furan of the Austrian population. Food Addit. Contam. Part A Chem. Anal. Control Expo. Risk Assess..

[16] European Commission (2017). Commission Regulation (EU) 2017/2158 of 20 November 2017 Establishing Mitigation Measures and Benchmark Levels for the Reduction of the Presence of Acrylamide in Food.

[17] Mogol B.A., Gokmen V. (2016). Thermal process contaminants: Acrylamide, chloropropanols and furan. Curr. Opin. Food Sci..

[18] Hamzahoglu A., Gökmen V. (2020). 5-Hydroxymethylfurfural accumulation plays a critical role on acrylamide formation in coffee during roasting as confirmed by multiresponse kinetic modelling. Food Chem..

[19] Mao L., Miao S., Yuan F., Gao Y. (2018). Study on the textural and volatile characteristics of emulsion filled protein gels as influenced by different fat substitutes. Food Res. Int..

[20] Sánchez R., Martín-Tornero E., Lozano J., Arroyo P., Meléndez F., Martín-Vertedor D. (2022). Evaluation of the olfactory pattern of black olives stuffed with flavored hydrocolloids. LWT-Food Sci. Technol..

[21] Stone H., Bleibaum R., Thomas H.A. (2020). Sensory Evaluation Practices.

[22] Anwar H., Anwar T., Murtaza S. (2023). Review on food quality assessment using machine learning and electronic nose system. Biosens. Bioelectron. X.

[23] Barea-Ramos J.D., Cascos G., Mesías M., Lozano J., Martín-Vertedor D. (2022). Evaluation of the Olfactory Quality of Roasted Coffee Beans Using a Digital Nose. Sensors.

[24] Ali M.M., Hashim N., Aziz S.A., Lasekan O. (2020). Principles and recent advances in electronic nose for quality inspection of agricultural and food product. Trends Food Sci. Technol..

[25] Peris M., Escuder-Gilabert A. (2009). A 21st century technique for food control: Electronic noses. Anal. Chim. Acta.

[26] Sánchez R., Pérez-Nevado F., Martillanes S., Montero-Fernández I., Lozano J., Martín-Vertedor D. (2023). Machine olfaction discrimination of Spanish-style green olives inoculated with spoilage mold species. Food Control.

[27] Montero-Fernández I., Marcía-Fuentes J.A., Cascos G., Saravia-Maldonado S.A., Lozano J., Martín-Vertedor D. (2022). Masking Effect of Cassia grandis Sensory Defect with Flavoured Stuffed Olives. Foods.

[28] Barea-Ramos J.D., Santos J.P., Lozano J., Rodríguez M.J., Montero-Fernández I., Martín-Vertedor D. (2023). Detection of Aroma Profile in Spanish Rice Paella during Socarrat Formation by Electronic Nose and Sensory Panel. Chemosensors.

[29] Sánchez R., Martín-Tornero E., Lozano J., Boselli E., Arroyo P., Meléndez F., Martín-Vertedor D. (2021). E-Nose discrimination of abnormal fermentations in Spanish-Style Green Olives. Molecules.

[30] Pérez-Nevado F., Cabrera-Bañegil M., Repilado E., Martillanes S., Martín-Vertedor D. (2018). Effect of different baking treatments on the acrylamide formation and phenolic compounds in Californian-style black olives. Food Control.

[31] Long Y., Zhu M., Ma Y., Huang Y., Gan B., Yu Q., Xie J., Chen Y. (2023). Variation of bioactive compounds and 5-hydroxumethyl furfural in coffee beans during the roasting process using kinetics approach. Food Chem. Adv..

[32] Celdrán A.C., Oates M.J., Molina Cabrera C., Pangua C., Tardaguila J., Ruiz-Canales A. (2022). Low-Cost Electronic Nose for Wine Variety Identification through Machine Learning Algorithms. Agronomy.

[33] Bressani A.P.P., Batista N.N., Ferreira G., Martinez S.J., Simao J.B.P., Dias D.R., Schwan R.F. (2021). Characterization of bioactive, chemical, and sensory compounds from fermented coffees with different yeasts species. Food Res. Int..

[34] da Silva O.E.C., da Luz J.M.R., de Castro M.G., Filgueiras P.R., Guarçoni R.C., de Castro E.V.R., da Silva M.d.C.S., Pereira L.L. (2022). Chemical and sensory discrimination of coffee: Impacts of the planting altitude and fermentation. Eur. Food Res. Technol..

[35] Ribeiro J.S., Teófilo R.F., Salva T.d.J., Augusto F., Ferreira M.M.C. (2023). Exploratory and discriminative studies of commercial processed Brazilian coffees with different degrees of roasting and de-caffeinated. Braz. J. Food Technol. Camp..

[36] Binello A., Cravotto G., Menzio J., Tagliapietra S. (2021). Polycyclic aromatic hydrocarbons in coffee samples: Enquiry into processes and analytical methods. Food Chem..

[37] Zhai Y., Xia X., Deng S., Cui H., Hayat K., Zhang X., Ho C.T. (2023). Reduced asynchronism be-tween regenerative cysteine and fragments of deoxyosones promoting formation of sulfur-containing compounds through extra-added xylose and elevated temperature during thermal processing of 2 threityl-thiazolidine-4-carboxylic acid. Food Chem..

[38] Costa A.M.d.S., Soares K.L., Silveira L.d.S., Filho A.C.V., Pereira L.L., Osorio V.M., Fronza M., Scherer R. (2024). Influence of maturation and roasting on the quality and chemical composition of new conilon coffee cultivar by chemometrics. Food Res. Int..

[39] García-Lomillo J., González-SanJosé M.L. (2019). Pyrazines in Thermally Treated Foods. Encyclopedia of Food Chemistry.

[40] Farah A., de Paula Lima J. (2019). Consumption of Chlorogenic Acids through Coffee and Health Implications. Beverages.

[41] Liu Q., Zhou P., Luo P., Wu P. (2023). Occurrence of furfural and its derivatives in coffee products in China and estimation of dietary intake. Foods.

[42] Seninde D.R., Chambers E. (2020). Coffee Flavor: A Review. Beverages.

[43] Caporaso N., Whitworth M.B., Cui C., Fisk I.D. (2018). Variability of single bean coffee volatile compounds of Arabica and robusta roasted coffees analysed by SPME-GC-MS. Food Res. Int..

[44] Marin K., Pozrl T., Zlatic E., Plestenjak A. (2008). A new aroma index to determine the aroma quality of roasted and ground coffee during storage. Food Technol. Biotechnol..

[45] Calda-Estada S.J., Utrilla-Vázqueza M., Vallejo-Cadona A., Roblero-Pérez D.B., Lu-go-Cervantes E. (2020). Thermal properties and volatile compounds profile of commercial dark-chocolates from different genotypes of cocoa beans (*Theobroma cacao* L.) from Latin America. Food Res. Int..

[46] Correia R.M., Loureiro L.B., Rodrigues R.R.T., Costa H.B., Oliveira B.G., Filgueiras P.R., Thompson C.J., Lacerda V., Romao W. (2016). Chemical profiles of Robusta and Arabica coffee by ESI(-)FT-ICR MS and ATR-FTIR: A quantitative approach. Anal. Methods.

[47] Cerny C., Schlichtherle-Cerny H., Gibe R., Yuan Y. (2021). Furfuryl alcohol is a precursor for fur-furylthiol in coffee. Food Chem..

[48] Chapko M.J., Seo H.S. (2019). Characterizing product temperature-dependent sensory perception of brewed coffee beverages: Descriptive sensory analysis. Food Res. Int..

[49] Lachenmeier D.W., Schwarz S., Teipel J., Hegmanns M., Kuballa T., Walch S.G., Breitling-Utzmann C.M. (2019). Potential antagonistic effects of acrylamide mitigation during coffee roasting on furfuryl alcohol, furan and 5-hydroxymethylfurfural. Toxics.

[50] Mesías M., Morales F.J. (2016). Acrylamide in coffee: Estimation of exposure from vending machines. J. Food Compos. Anal..

[51] Bagdonaite K., Derler K., Murkovic M. (2008). Determination of acrylamide during roasting of coffee. J. Agric. Food Chem..

[52] Park S.H., Jo A., Lee K.G. (2021). Effect of various roasting, extraction and drinking conditions on furan and 5-hydroxymethylfurfural levels in coffee. Food Chem..

[53] Abraham K., Gürtler R., Berg K., Heinemeyer G., Lampen A., Appel K.E. (2011). Toxicology and risk assessment of 5-hydroxymethylfurfural in food. Mol. Nutr. Food Res..

[54] Martín-Tornero E., Sánchez R., Lozano J., Martínez M., Arroyo P., Martín-Vertedor D. (2021). Characterization of polyphenol and volatile fractions of Californian-style black olives and innovative application of e-nose for acrylamide determination. Foods.

[55] Mesías M., Barea-Ramos J.D., Lozano J., Morales F.J., Martín-Vertedor D. (2023). application of an electronic nose technology for the prediction of chemical process contaminants in roasted almonds. Chemosensors.

[56] Aghdamifar E., Sharabiani V.R., Taghinezhab E., Szymanek M., Dziwulska-Hunek A. (2023). E-nose as a non-destructive and fast method for identification and classification of coffee beans based on soft computing models. Sens. Actuators B Chem..

[57] Adelina N.M., Wang H., Zhang L., Zhao Y. (2021). Comparative analysis of volatile profiles in two grafted pine nuts by headspace-SPME/GC-MS and electronic nose as responses to different roasting conditions. Food Res. Int..

[58] Dong W., Hu R., Long Y., Li H., Zhang Y., Zhu K., Chu Z. (2019). Comparative evaluation of the volatile profiles and taste properties of roasted coffee beans as affected by drying method and detected by electronic nose, electronic tongue and HS-SPME-GC-MS. Food Chem..

[59] Rodríguez J., Durán C., Reyes A. (2009). Electronic nose for quality control of Colombian coffee through the detection of defects in “Cup Tests”. Sensors.

[60] Zhang K., Cheng J., Hong Q., Dong W., Chen X., Wu G., Zhang Z. (2022). Identification of changes in the volatile compounds of robusta coffee beans during drying based on HS-SPME/GC-MS and E-nose analyses with the aid of chemometrics. LWT.

